# Prevalence of Amblyogenic Risk Factors Among School Children in India Using the Spot Vision Screener

**DOI:** 10.7759/cureus.66977

**Published:** 2024-08-16

**Authors:** Siddharam S Janti, Veera Manasa Alluri, Raghavendra Rao Kolavai, Kalpana Mali, Sahithi Eereti, Bhushan Kamble

**Affiliations:** 1 Ophthalmology, All India Institute of Medical Sciences, Bibinagar, Bibinagar, IND; 2 Medicine, All India Institute of Medical Sciences, Bibinagar, Bibinagar, IND; 3 Pharmacology, All India Institute of Medical Sciences, Bibinagar, Bibinagar, IND; 4 Community and Family Medicine, All India Institute of Medical Sciences, Bibinagar, Bibinagar, IND

**Keywords:** anisometropia, strabismus, refractive error, spot vision screener, amblyopia

## Abstract

Introduction: Amblyopia is a common cause of preventable visual impairment in children, affecting 1% to 6% globally. This study assesses amblyopia prevalence and risk factors among school children in rural Telangana, India, using the Spot Vision Screener (Welch Allyn, Inc., Skaneateles Falls, New York, USA), a portable, noninvasive device recommended for automated vision screening.

Methods: A cross-sectional study was conducted on 714 schoolchildren aged 5-10 years. Screening was performed using the Spot Vision Screener, evaluating refractive errors, ocular alignment, and other amblyopia risk factors. Children identified with potential amblyogenic factors were referred for comprehensive ophthalmological evaluation to confirm diagnosis.

Results: Out of 714 children screened, 84 were referred by the Spot Vision Screener for further evaluation. Subsequent examination by ophthalmologists confirmed amblyopia in 65 children, resulting in a prevalence of 9.10%. Myopic refractive error was the most prevalent (69.23%), followed by astigmatism (21.53%) and hypermetropia (9.23%) among amblyopia cases.

Conclusion: The Spot Vision Screener proved to be a reliable tool for identifying amblyopia risk factors in school children, facilitating early detection and referral for appropriate management. This study underscores the importance of implementing effective vision screening programs in rural settings to mitigate preventable childhood blindness.

## Introduction

Amblyopia is a neurodevelopmental abnormality that results in physiological alterations in the visual pathways which can be unilateral or rarely bilateral, a decrease in best corrected visual acuity (BCVA) caused by form vision deprivation and/or abnormal binocular interaction, for which there is no identifiable pathology of the eye or visual pathway. This could be because of strabismus, anisometropia, stimulus deprivation, etc. [1]. It remains the most common cause of preventable visual loss in children, affecting 1% to 6% of all children, with nearly 100 million children suffering from it [2-4]. The prevalence of amblyopia in rural Telangana is 1.2%. If left untreated, amblyopia could result in complete vision loss in one or both eyes.

Traditional methods, such as auto-refractometer, are effective in identifying the causes of amblyopia. However, cooperation from the patient is important and requires more qualified manpower. Photoscreening devices and vision screening such as spot vision screener initiatives have been extensively studied and continue to be shown to be cost-effective, efficient, and effective methods for detecting risk factors for amblyopia [5], compared to traditional methods.

The Spot Vision Screener (Welch Allyn, Inc., Skaneateles Falls, New York, USA), which explores refraction status by recording the reflexes of both pupils simultaneously, is used in this study. It is a non-invasive, handheld, touch screen, portable rechargeable device. The measuring range is ±7.50 diopters (D) for spherical errors and ±3.00 D for cylindrical errors. The device warns the examiner about significant refractive errors, anisometropia, anisocoria, and strabismus [6].

Even though the American Association for Pediatric Ophthalmology and Strabismus (AAPOS) recommended photo screener use to detect amblyopic risk factors, it has not been implemented in developing countries because lack of studies to assess the effectiveness of these instruments. A Spot Vision Screener is a portable and cost-effective tool that can detect risk factors among school children in rural Telangana, in accordance with the 2013 AAPOS Vision Screening Committee guidelines. For children over 48 months of age, the guidelines define refractive errors as follows: astigmatism greater than 1.5D, hyperopia greater than 3.5D, anisometropia greater than 1.5D, and myopia greater than 1.5D. Non-refractive errors include a manifest squint greater than 8 prism diopters (PD) in the primary position and media opacity greater than 1mm.

## Materials and methods

This study adhered to the guidelines of the Helsinki Declaration and received approval from the local Ethics Committee of the hospital “All India Institute of Medical Sciences, Bibinagar” and the Institutional Review Board with approval code: AIIMSIBBNIIECISEPT/2023/320.

Study design and participants

This cross-sectional study included both male and female school-going children aged 5-10 years from rural Telangana, India. Children with non-vision-threatening conditions such as conjunctivitis, external hordeolum, and those unwilling to participate were excluded. Initially, a sample size of 369 children was calculated using the formula Z^2^PQ/e^2^, with a z-value of 1.96, a P-value of 4%, and an absolute precision of 2% based on a previous study. Ultimately, a total of 714 children were screened for risk factors of amblyogenic risk factors.

Procedure

Informed consent was obtained from parents or the school principal before conducting any assessments. All participants underwent screening using the Spot Vision Screener. The device was positioned approximately 3 feet from the subject, who focused on a display of twinkling light. Upon initiating data capture, the screen displayed the child's face and a spinning circle while providing distance information. The Spot Vision Screener recorded pupillary diameter, ocular alignment, and estimated binocular refraction, with data collection taking approximately two seconds per child.

Evaluation and referral criteria

Following the Spot Vision Screener assessment, a recommendation for a complete eye examination was made for children exhibiting refractive errors such as astigmatism ≥+1.00 D, myopia ≤−0.75 D, hyperopia ≥+2.00 D, and non-refractive issues like significant media opacity or strabismus >8 PD, in accordance with AAPOS guidelines. Children identified with these conditions were re-examined by an optometrist to confirm the diagnosis of refractive error and associated amblyopia. Those diagnosed with refractive error, strabismus, or visual deprivation amblyopia were referred to a tertiary healthcare center for further management.

## Results

A total of 714 school children (mean age 7.7 years±1.7) underwent screening for amblyopia. Out of 714 screened, 84 (11.8%) school students had amblyogenic risk factors by Spot Vision Screener test. Optometrist examined these screen-positive students for confirmation of amblyopia, 65 (9.1%) had amblyopia by optometrist examination. Among them, 35 out of 390 males (8.97%) and 30 out of 324 females (9.25%) were diagnosed with amblyopia. There is no significant association between gender and amblyopia (p-value>0.05) (Table 1). Additionally, the association of amblyopia with age was examined. The results showed that 36 out of 414 children aged 5-8 years (8.69%) and 29 out of 300 children aged 8-10 years (9.66%) had amblyopia and we did not find a significant association with the occurrence of amblyopia (p-value>0.05) (Table 2).

**Table 1:** Association of amblyopia with gender (n=714)

	Amblyopia		Total (%)	Significance
Gender	Yes (%)	No (%)		χ2=0.04; p=0.84; df=1
Male	35 (8.97)	355 (91.03)	390 (100)	
Female	30 (9.25)	294 (90.75)	324 (100)	

**Table 2:** Association of amblyopia with age (n=714)

	Amblyopia		Total (%)	Significance
Age of the child	Yes	No		χ2=0.44; p=0.51; df=1
5-8 years	36 (8.69%)	378 (91.31%)	414 (100)	
8-10 years	29 (9.66%)	271 (90.34%)	300 (100)	

Among the 65 participants diagnosed with amblyopia, there was a near-equal gender distribution, with 53.84% male and 46.15% female (Table 3). The primary cause of amblyopia was refractive error, accounting for 93.84% of cases, followed by strabismus (4.61%) and visual deprivation amblyopia (1.5%) (Table 4). Within the category of refractive errors, myopia was the most common, affecting 69.23% of those with amblyopia, while hypermetropia and astigmatism accounted for 9.23% and 21.53% of cases, respectively (Table 5).

**Table 3:** Distribution of study participants with amblyopia as per gender (n=65)

Gender	Number	Percentage
Male	35	53.84
Female	30	46.15

**Table 4:** Distribution of study participants with amblyopia as per cause (n=65)

Types/causes of amblyopia	Number	Percentage
Refractive	61	93.84
Strabismus	3	4.61
Visual deprivation amblyopia	1	1.5

**Table 5:** Distribution of study participants with amblyopia as per type of refractive error (n=65)

Different types of refractive error	Number	Percentage
Myopia	45	69.23
Hypermetropia	6	9.23
Astigmatism	14	21.53

Regarding the performance of the Spot Vision Screener in detecting amblyopia, it correctly identified 65 true positive cases (9.1%) and 630 true negative cases (88.2%) out of 714 children. However, it also produced 19 false positive results (2.7%) and nine false negative results (1.3%) (Table 6). Compared to optometrist examination results, the Spot Vision Screener had a sensitivity of 87.8% and specificity of 97%, demonstrating its high accuracy in identifying amblyopia in the field (Table 7).

**Table 6:** Distribution of study participants as per amblyopia screening status (n=714)

Students with amblyopia by Spot Vision Screener (%)	True positive (%)	False positive (%)	False negative (%)	True negative (%)	Total number of children screened (%)
84 (11.8)	65 (9.1)	19 (2.7)	9 (1.3%)	630 (88.2)	714 (100)

**Table 7:** Distribution of study participants as per Spot Vision Screener and ophthalmic examination (n=714)

Spot Vision Screener		Ophthalmologists examination		
		Amblyopia+ (%)	Amblyopia- (%)	Total
	Amblyopia+	65 (87.8)	19 (3)	84 (11.8)
	Amblyopia-	9 (12.2)	621 (97)	630 (88.2)
	Total	74 (100)	640 (100)	714 (100)
	Sensitivity	87.8%		
	Specificity	97%		

## Discussion

This cross-sectional study, conducted between October and November 2023, evaluated the prevalence, demographic characteristics, and risk factors associated with amblyopia among children aged 5-10 years in rural Telangana, India. A total of 714 school-going children were screened using the Spot Vision Screener, with diagnoses confirmed by ophthalmologists. The study aimed to provide insights into the burden of amblyopia and the effectiveness of screening methods in a resource-limited setting.

Our study identified 84 children with amblyogenic risk factors through initial screening. Ophthalmological examination confirmed amblyopia in 65 of these children, resulting in a prevalence rate of 9.10% in identifying amblyogenic risk factors whereas Rodriguez and Castro González reported 1.8% [7], Dutta Chowdhury et al. found 2.9% in West Bengal [8], Kalikivayi et al. reported 1.1% [9], and Anjaneyulu and Reddy [10] observed 6.6% of prevalence in amblyopia.

The higher prevalence in our study compared to urban settings in India is also noteworthy. For example, a study conducted in Bangalore reported a prevalence of approximately 3.7% among children aged 5-15 years [11]. Urban areas may benefit from better access to healthcare services and more consistent screening programs, potentially contributing to lower prevalence rates. This highlights the impact of healthcare accessibility and socioeconomic factors on the prevalence of amblyopia.

When comparing with rural settings in other countries, our prevalence rate is consistent with findings from rural areas in other developing nations. For instance, a study in rural China reported a prevalence of about 8.2% among children aged 4-6 years [12]. This suggests that rural areas, regardless of the country, face similar challenges related to access to eye care and early detection of amblyopia.

Several factors may contribute to this disparity, including increased screen time and online classes during the pandemic, which have been linked to a rise in visual impairments among children [13]. Among the 714 children screened, 35 out of 390 males (8.97%) and 30 out of 324 females (9.25%) were diagnosed with amblyopia. The prevalence was slightly higher in males (53.84%) than females (46.15%). This contrasts with findings from Dutta Chowdhury et al. [8], who reported a higher prevalence in males (57.1%), while studies by Daigavane and Prasad [14], Park et al. [15] and Anjaneyulu and Reddy [10] observed higher prevalence in females.

Our study also found that amblyopia was more common in the 5-8 year age group (36 children) compared to the 8-10 year age group (29 children), with a p-value of 0.51, indicating that age was not significantly associated with the occurrence of amblyopia. This contrasts with Dutta Chowdhury et al. [8] who found a higher prevalence in children aged 11-16 years, and Gupta et al. [16,17] reported a higher prevalence in the 5-10 year age group.

Among the 65 children with amblyopia, the primary causative factor was refractive error (93.84%), followed by strabismus (4.61%) and visual deprivation amblyopia (1.5%). These findings are consistent with Shah et al., who reported a higher prevalence of anisometropic amblyopia (36.20%) and strabismic amblyopia (25.86%). In contrast, Attebo et al. [16] found a higher prevalence of anisometropic amblyopia (50%) compared to strabismic amblyopia (19%). Dutta Chowdhury et al. [8] reported anisometropic amblyopia (34%) and strabismic amblyopia (29.5%), while Daigavane and Prasad [14] and Gupta et al. [17] also identified anisometropic amblyopia as the most common type, with 53.8% and 72%, respectively.

Anisometropia, characterized by unequal refractive power between the eyes, is a well-established risk factor for amblyopia [14]. The predominance of unilateral cases supports the need for early and effective screening strategies to prevent irreversible vision loss. Out of 61 cases of refractive errors in amblyopia, myopia was the most common, affecting 69.23% of children, while hypermetropia and astigmatism accounted for 9.23% and 21.53% of cases, respectively. This contrasts with Menon et al. [18] who reported that amblyopia due to hypermetropia was the highest (51.7%).

The Spot Vision Screener demonstrated a sensitivity of 87.86% and specificity of 97.03%, comparable to or exceeding the efficacy reported in other studies. For example, Barugel et al. [19] reported a sensitivity of 82.35% and a specificity of 91.67%, while Panda et al. [20] found a sensitivity of 93.3% and a specificity of 96.9%. Similarly, Misra et al. [21] reported a sensitivity of 91.7% and specificity of 93.4%. The high sensitivity and specificity of the Spot Vision Screener in our study suggest it is an effective tool for identifying amblyogenic risk factors and facilitating timely intervention. However, variability in screening efficacy, as noted in reports by Kemper et al. [22] and Forcina et al. [23], underscores the importance of considering potential false positives and negatives in screening programs. Due to its high specificity, the Spot Vision Screener minimizes unnecessary hospital care costs, and its high sensitivity allows for the confident detection of amblyopia. It can be used effectively in pediatric settings where ophthalmologists may not be available, with minimal training required for the device. Our study highlights the importance of educating parents and teachers about amblyopia and its impact on a child's future. Annual screening of school children could significantly reduce the incidence and prevalence of amblyopia.

The limitations of the study are as follows: First, the cross-sectional design restricts the ability to establish causality or track changes over time, making it challenging to understand the progression of amblyopia or the long-term effects of screening interventions. Second, the research was conducted in a specific rural area of Telangana, which may not accurately reflect amblyopia prevalence or risk factors in other regions of India or globally. Additionally, the study did not fully account for all possible confounding variables, such as socioeconomic status, parental education, and healthcare access, which could also influence amblyopia rates and screening results. Addressing these limitations in future research could provide a more comprehensive understanding of amblyopia and its influencing factors across diverse populations.

## Conclusions

This study underscores the critical need for regular vision screening, particularly in rural areas where access to eye care services is often limited. By identifying amblyopia and its risk factors early, we can significantly reduce the incidence of preventable vision impairment among children. The high prevalence of amblyopia observed in rural Telangana highlights the importance of implementing consistent screening programs to address this public health concern and ensure timely intervention for affected children.

The Spot Vision Screener has proven to be an invaluable tool in this context, offering a noninvasive and efficient method for detecting amblyopic risk factors. Its effectiveness in resource-limited settings demonstrates its potential to transform vision screening practices, making it easier to identify and manage amblyopia in underserved populations. The integration of such screening technologies into routine health practices could greatly improve outcomes and reduce the long-term impact of amblyopia on children's visual health.
